# Antimicrobial Susceptibility among Pathogens Isolated in Early- versus Late-Onset Ventilator-Associated Pneumonia

**DOI:** 10.3390/idr13020038

**Published:** 2021-04-27

**Authors:** Hend Ben Lakhal, Aymen M’Rad, Thierry Naas, Nozha Brahmi

**Affiliations:** 1 Service de Reanimation, Centre Hospitalier de Chartres, 4, Rue Claude-Bernard, 28630 Le Coudray, France; 2 Service de Reanimation, Centre d’Assistance Médicale Urgente (CAMU) de Tunis, 50 Rue Abou Kacem Chebbi, Tunis 1089, Tunisia; aymenmrad140519@gmail.com (A.M.); n.brahmi@gmail.com (N.B.); 3 Bacteriology-Hygiene Unit, Assistance Publique/Hôpitaux de Paris, Bicêtre Hospital, 94270 Le Kremlin-Bicêtre, France; thierry.naas@aphp.fr; 4 Team ReSIST, INSERM U1184, School of Medicine Université Paris-Saclay, LabEx LERMIT, 94270 Le Kremlin-Bicêtre, France

**Keywords:** VAP, causative pathogens, antibiotic therapy, MDR

## Abstract

Ventilator-associated pneumonia (VAP) is associated with increased hospital stay and high morbidity and mortality in critically ill patients. The aims of this study were to (i) determine the incidence of multidrug-resistant (MDR) pathogens in the first episodes of VAP and to assess potential differences in bacterial profiles of subjects with early- versus late-onset VAP. This was a retrospective cohort study over a period of 18 months including all patients who had a first episode of VAP confirmed by positive bacterial culture. Subjects were distributed into two groups according to the number of intubation days: early-onset VAP (<5 days) or late-onset VAP (≥5 days). The primary endpoint was the nature of causative pathogens and their resistance profiles. Sixty patients were included, 29 men and 31 women, with an average age of 38 ± 16 years. The IGS 2 at admission was 40.5 [32–44] and APACHE was 19 [15–22]. Monomicrobial infections were diagnosed in 77% of patients (*n* = 46). The most frequently isolated bacteria were *A. baumannii*, 53% (*n* = 32); *P. aeruginosa* in 37% (*n* = 22); Enterobacterales in 28% (*n* = 17) and *S. aureus* in 5% (*n* = 3). Ninety-seven percent of the bacteria were MDR. The VAP group comprised 36 (60%) episodes of early-onset VAP and 24 (40%) episodes of late-onset VAP. There was no significant difference in the distribution of the bacterial isolates, nor in terms of antibacterial resistances between early- and late-onset VAPs. Our data support recent observations that there is no microbiological difference in the prevalence of potential MDR pathogens or in their resistance profiles associated with early- versus late-onset VAPs, especially in countries with high rates of MDR bacteria.

## 1. Introduction

Ventilator-associated pneumonia (VAP) refers to “any pneumonia occurring in a patient whose respiration is assisted by a machine, either invasively via an endotracheal tube or a tracheotomy, or non-invasively via a face mask or other procedure, within 48 h of the onset of infection” [1]. It is the leading cause of hospital-associated infection in intensive care units [2]. Its incidence serves as a quality indicator for intensive care units (ICUs) and it ranges from 15–45% and 8–28% in mechanically ventilated patients [2,3,4]. VAP represents a real public health problem because it leads to an increase in the duration of mechanical ventilation (MV) and hospital stay [3,5], additional costs [6,7] and increased mortality in the order of 13% [8]. Classically, a dichotomy was made between early- and late-onset VAP on the basis of bacteriological data, which imply a different probabilistic antibiotic therapy according to the delay of occurrence [9]. However, in recent years, this notion has been questioned as several studies carried out in different countries have shown no difference in the bacteriological profile between early and late VAP.

Common causative pathogens of VAP include Gram-negative bacteria (GNB) such as *Pseudomonas aeruginosa*, *Escherichia coli*, *Klebsiella pneumoniae* and *Acinetobacter* species, and Gram-positive bacteria (GPB) such as *Staphylococcus aureus* [10]. The type of causative pathogen and its rate of drug resistance may vary depending on the country, the region and the hospital [10]. A dramatic increase in third-generation cephalosporin resistance among GNB and carbapenem resistance in non-fermenters has been witnessed in a Tunisian hospital [11,12,13]. Carbapenem resistance leads to resistance to almost all β-lactams and is generally associated with other resistance mechanisms affecting other families of antibiotics, which illustrates a typical example of a multidrug-resistant (MDR) or even pan-drug-resistant (PDR) phenotype [11,12]. In North Africa, the prevalence of carbapenem-resistant Enterobacterales (CRE) isolates in the hospital setting ranged from 2.3% to 67.7% [14] and reached almost 90% for carbapenem-resistant *A. baumannii* [13].

The main objective of this study is to determine the incidence of MDR bacteria in the first episodes of VAP and to investigate possible differences in the bacteriological profile between early and late VAPs.

## 2. Materials and Methods

### 2.1. Study Design and Inclusion Criteria

We performed a prospective observational study in a 10-bed medical and toxicological ICU over an 18-month period, from January 2013 to June 2014. We included all patients, aged more than 14 years, under mechanical ventilation for at least 48 h and who developed a first episode of VAP. The diagnosis of VAP was suspected with the criteria of Johanson et al. [15]: a new or progressive pulmonary infiltrate on the chest X-ray associated with two of the following criteria: temperature above 38 °C or below 36.5 °C with no other cause, white cell count above 12,000/mm^3^ or below 4000/mm^3^, purulent respiratory secretions or worsening of the hemodynamic and/or respiratory state without any other obvious reason. VAP confirmation was defined by the quantitative culture of blinded protected specimens with at least 10^3^ cfu/mL [16]. Patients aged under 14 years and those who were treated for suspected VAP without bacteriological confirmation (negative culture or with no significant count in quantitative culture) were excluded. Only the first episode was included, subsequent episodes were not analyzed. Informed consent was obtained from the patients, or close relatives, and the project had clearance from the local ethics committee.

### 2.2. Bacterial Identification, Antimicrobial Susceptibility Testing

Bacteria were identified using standard conventional methods in conjunction with matrix-assisted laser desorption/ionization time of flight mass spectrometry (MALDI-TOF MS) (Bruker Daltonics, Hamburg, Germany). Susceptibility testing was performed by the disc diffusion method [17]. MICs of carbapenems were determined using E-Test (bioMérieux, Marcy-l’-Etoile, France). The presence of an ESBL was investigated by a synergy image detection using the double-disc synergy test (DDST) performed with ceftazidime and ticarcillin/clavulanic acid discs on cloxacillin (200 mg/L)-containing Mueller–Hinton (MH) agar plates, which partially inhibits cephalosporinase activity [17]. European Committee on Antimicrobial Susceptibility Testing (EUCAST) methods and interpretation criteria were used for all antimicrobial agents [18].

### 2.3. Data Collection Protocol

Chart review data included demographics, comorbid conditions, prior antimicrobial exposure (one week before admission), physical examination findings, laboratory and microbiology data, chest radiograph reports and severity scores at admission, including Simplified Acute Physiology score (SAPS II), Acute Physiology and Chronic Health Evaluation II score (APACHE II), Sepsis-related Organ Failure Assessment score (SOFA) and Clinical Pulmonary Infection Score (CPIS) [19], together with the reason for ICU admission and for starting mechanical ventilation. We extracted these parameters at the time of enrolment in the study.

### 2.4. Group Definitions and Evaluation Criteria/Clinical Outcome

Subjects were distributed into 2 groups according to the number of intubation days: early-onset VAP (<5 days) or late-onset VAP (≥5 days).

The primary endpoint of the present study was the nature of the isolated pathogens and their antimicrobial resistance pattern. Multidrug resistance was considered when species showed resistance to at least three groups of antibiotics.

The secondary criteria were (i) the occurrence of complications such as: severe sepsis or septic shock and acute respiratory distress syndrome (ARDS) according to the Berlin definition of 2012 [20]; (ii) 3-day, 7-day, 14-day and 28-day mortality after the occurrence of VAP; (iii) the duration of mechanical ventilation; and (iv) ICU length of stay.

### 2.5. Statistical Analysis

Categorical variables were expressed as absolute and relative (%) frequencies, whereas continuous ones were expressed as the median and the standard deviation (SD) or the median and the interquartile range (IQR). Differences between groups of early-onset and late-onset VAP were analyzed. Continuous variables were compared using a Student’s *t*-test for normally distributed variables and the Mann–Whitney U test for non-normally distributed variables. Categorical variables were compared using the χ^2^ and Fisher’s exact tests, where appropriate. Differences between groups were considered significant if the *p* value was <0.05. Collected data were entered and analyzed using SPSS 20.0 software (Armonk, NY, USA).

## 3. Results

### 3.1. Clinical History of Included Patients

A total of 60 subjects who met the criteria for VAP with confirmed microbiology results were included in the current analysis (Table 1). Subjects were stratified into early-onset (*n* = 36) and late-onset VAP (*n* = 24). Both groups had a similar sex ratio and age and similar rates of comorbidity conditions. In addition, the mean CPIS was 7 ± 1 and the mean duration of mechanical ventilation before occurrence of the first episode of VAP was 5.3 ± 3 days. There were no significant differences in CPIS between the early-onset and late-onset VAP subjects. However, the APACHE II, SAPS II and SOFA scores were significantly higher in the late-onset VAP subjects compared to early-onset VAP (21 vs. 17, *p* = 0.002; 41 vs. 36, *p* = 0.049; and 8 vs. 5.5, *p* = 0.037, respectively).

We found that patients admitted with a diagnosis of acute poisoning were more likely to develop early-onset VAP (*p* ˂ 0.0001). Nevertheless, patients with respiratory failure were more likely to develop late-onset VAP (*p* = 0.004). Acute exacerbation of COPD was the most frequent cause of acute respiratory failure (6/12), followed by community-acquired pneumonia (4/12) and submersion (2/12). Neurological diagnoses were encephalitis (one patient), seizures (one patient), severe head trauma (one patient) and intracranial haemorrhage (one patient).

Prior antibiotic use was significantly higher among late-onset VAP patients (92% vs. 52%, *p* = 0.002). Antimicrobial therapy included cefotaxime (51%), amoxicillin–clavulanate (44%), metronidazole (24%), ofloxacin (12%), aminoglycosides (8%), piperacillin–tazobactam (2.4%), imipenem (2.4%) and cotrimoxazole (2.4%).

### 3.2. Microbial and Antibacterial Resistance Patterns

Among the 60 VAP cases, 46 (77%) were monomicrobial (one bacterial species) and 14 (23%) were polymicrobial (two bacterial species) (Table 2). The most common isolated organisms were *A. baumannii* (53%), *P. aeruginosa* (37%) (Table 3) and *Enterobacterales* (28%), including *K. pneumoniae* (*n* = 11), *Enterobacter cloacae* complex (*n* = 2), *E. coli* (*n* = 1), *Proteus mirabilis* (*n* = 1), *Providencia stuartii* (*n* = 1) and *Serratia marcescens* (*n* = 1).

*A. baumannii* showed resistance to all groups of drugs, including carbapenems, except colistin (Table 4). Among the 22 P. aeruginosa isolates, 44% were resistant to carbapenems (Table 4). All Enterobacterales remained susceptible to carbapenems, but 71% of them were resistant to expanded cephalosporins by the production of an ESBL (Table 4). No colistin resistance was observed among the studied bacterial isolates and 97% of the tested isolates were MDR bacteria.

The frequency of the occurrence of these most frequent pathogens and their antimicrobial resistance pattern were similar regardless of the time of VAP occurrence (Table 5).

## 4. Discussion

The aim of this study was to evaluate incidence of MDR bacteria in the first episodes of VAP and to seek for a possible difference between early and late VAP concerning isolates and their resistance patterns. We found that the distribution and frequencies of isolated pathogens were similar in the two groups. Moreover, the incidence of MDR bacteria was alarming in the first episodes of VAP.

Nosocomial infections are a global public health problem, resulting in higher mortality [21]. Acquisition of antibiotic resistance mechanisms is a natural process, that is amplified by overuse and misuse of antibiotics and inadequate or the absence of epidemiological surveillance in human and veterinary medicine, which has led to the dissemination of these MDR bacteria, especially of *Enterococcus faecium*, *S. aureus*, *K. pneumoniae*, *A. baumannii*, *P. aeruginosa* and *Enterobacter* spp. (ESKAPE bacteria). This situation brings us back to the pre-antibiotic era and thus threatens many achievements of modern medicine that rely on effective antibiotic therapies.

In the United States, the number of healthcare-associated infections caused by MDR bacteria was estimated at more than 2 million in 2011, with 37,000 deaths [22]. The ECDC report by Cassini et al. [23] has precisely determined that 672,000 infections occurred in the EU in 2015, that 33,000 people died as a consequence of their infection and that these infections have led to the loss of 874,000 years of good quality of life [23]. Moreover, 40% of the deaths were related to MDR-GNB. *Enterobacterales* account for the majority of antibiotic-resistant bacteria and are increasing the most rapidly.

Nosocomial infections are common in ICUs, because of medical invasive devices [24]. VAP still remains as the leading infection among critically ill patients, with an estimated incidence between 8% and 28% [1,2,3,4]. It is associated with an excess of costs, in addition to prolonged mechanical ventilation [5,6,7]. The attributable mortality is about 13% [8]. The administration of accurate and timely initial empirical antibiotic therapy has been shown to have a major impact on mortality [25]. In a meta-analysis including 10 studies, Kuti et al. found that inappropriate antibiotic therapy significantly increased attributable mortality (odds ratio = 2.34, 95% CI [1.51–3.63], *p* = 0.0001). Thus, intensivists are facing the dilemma to treat rapidly by broad-spectrum empiric antibiotic therapy and to limit its use, as it is a source of resistance emergence [26].

Considering the time of VAP onset (3rd to 7th day of mechanical ventilation), several studies suggested that pathogens responsible for early VAP were essentially normal oropharynx flora (such as *H. influenzae*, *S. pneumoniae*, methicillin-sensitive *S. aureus* and susceptible *Enterobacterales*). Late VAPs were due to MDR bacteria, including *P. aeruginosa*, *A. baumannii*, methicillin-resistant *S. aureus* and resistant *Enterobacterales* [1,27,28,29]. The main reason leading to differences in antimicrobial resistance patterns was related to prior antibiotic use among late-onset VAP patients [1]. As a result of these studies, the American Thoracic Society (ATS) guidelines, published in 2005, classified patients into two groups on the basis of time of onset of VAP (early onset vs. late onset) and the presence of risk factors for infection with MDR bacteria. In the case of early VAP, susceptible organisms should be considered in the absence of risk factors for MDR bacteria (recent hospitalization, haemodialysis, home care, etc.) [9].

In the same year, Giantsou et al. assessed 408 cases of VAP diagnosed by bronchoalveolar lavage (BAL) and showed that both early-onset (<7 days) and late-onset VAP (>7 days) are caused by MDR bacteria even in the absence of risk factors [30]. Subsequently, similar studies confirmed that there was no difference between early and late VAP (regardless of VAP onset day) [31,32,33,34,35]. These results were explained by the local ecology of ICUs, in which the various studies were carried out and where the incidence of MDR bacteria was high. As a result, the new guidelines of the Centers for Disease Control and Prevention (CDC) from 2014 consider local ecology as a very important risk factor in the development of VAP caused by MDR bacteria, regardless of the time of onset [36].

In our study, we found that both early-onset and late-onset VAP was mainly caused by potentially MDR bacteria, most commonly *A. baumannii* and *P. aeruginosa*, and there was no difference concerning the resistance profile of the isolates. Our results, which are explained by the high prevalence of MDR bacteria in our ICU, were consistent with recent studies reported in the literature [37]. In their state-of-the-art review, Chastre and Fagon compiled microbiology data from 24 published studies that confirmed 1689 episodes of VAP involving 2490 isolates of pathogens. The most common pathogen was *P. aeruginosa*, accounting for 24% of isolates. The second most common was *S. aureus*, accounting for 20.4% of isolates, of which 56% were methicillin-resistant strains. The *Enterobacterales* were the third most common group of pathogens, accounting for 14.1% of isolates [1]. In the study by Ali et al., Gram negatives, such as *E. coli*, *P. aeruginosa*, *K. pneumoniae* and *A. baumannii*, accounted for most of the pathogens (74%), while *S. aureus* accounted for 20% [38]. In our study, *A. baumannii* was the most common pathogen, found in 84% (*n* = 53) of patients, followed by *P. aeruginosa* in 37% (*n* = 22) and *K. pneumoniae* in 18% (*n* = 11), while *S. aureus* represented only 5% of cases. Several recent studies have highlighted the increased level of the polymicrobial nature of VAPs. Fagon et al., in a prospective study, showed that 40% of all VAP specimens were polymicrobial, with more than one potential pathogen [39]. Similarly, Combes et al. found that polymicrobial infections were diagnosed in in 59 patients (48%), with two, three and four different bacteria found in 42 patients (34%), 10 patients (8%) and 7 patients (6%), respectively [40]. In 2013, Restrepo et al. showed that 50% of subjects in both early- and late-onset VAP groups had ≥2 pathogens [34]. Our culture results were monomicrobial in 77% of cases, while they were polymicrobial in 23% of VAP episodes. Finally, we have observed high resistance rates to different antibiotic classes, especially for *A. baumannii*, which was resistant to all β-lactams and fluoroquinolones in all patients. Similarly, we have observed high rates of ESBL producers, mainly *K. pneumoniae*. These results are in line with the epidemiology of MDR bacteria in Tunisia [12,13,14], and reflect epidemic situations in Tunisian ICUs, especially for carbapenem-resistant *P. aeruginosa* and *A. baumannii*. The increasing rates of MDR bacteria are mainly related to indwelling medical devices and selective pressure exerted by exposure to antimicrobial agents [41,42,43,44,45]. Our findings suggest that antimicrobial agents against *A. baumannii*, *P. aeruginosa* and *K. pneumoniae* should be prescribed empirically, at least in our institution, to patients suspected of having either early-onset or late-onset VAP. This may help to reduce the occurrence of inadequate or ineffective antimicrobial therapy, which has been associated with poorer outcomes. Prompt intravenous broad-spectrum antibiotic initiation, with the combination of imipenem and colistin at higher doses, regardless of the time of VAP onset, should be recommended. Aerosolized colistin may be an effective adjunctive therapy, as it improves clinical response and microbiological eradication, but does not affect overall mortality [44].

Our study presents some biases as the patients included in this study were from a very specialized ICU, caring for patients with severe toxicologic problems and frequent associated neurologic disorders. In addition, the study focused only on the first episode of VAP, due to an average follow-up period of 18 months, and a small number of patients was included, which is the consequence of a monocentric prospective study, which are also shortcomings of our study. Hence, it is difficult to extrapolate our results to other ICUs with different types of patients.

## 5. Conclusions

Our study demonstrates that in our ICU, whenever the onset time, VAPs were caused mainly by MDR bacterial pathogens, most commonly *A. baumannii* and *P. aeruginosa*. There were no significant differences in the prevalence nor in the resistance patterns of MDR pathogens associated with early-onset or late-onset VAPs. We suggest that initial empirical therapy with broad-spectrum antimicrobials for early-onset VAP should be considered in high-prevalence settings. Further prospective studies are needed to confirm these findings.

## Figures and Tables

**Table 1:** Baseline demographic and clinical characteristics of early-onset versus late-onset ventilator-associated pneumonia.

Clinical Data	Total Patients (*n* = 60)	Early-Onset VAP (*n* = 36)	Late-Onset VAP (*n* = 24)	*p*
Age, years (mean ± SD)	38 ± 16	35 ± 15	42 ± 18	1.25
Sex-ratio (male/female)	29/31	19/17	10/14	0.403
Underlying comorbidity				
-Psychiatric disorders, *n* (%)	20 (33)			
-COPD, *n* (%)	5 (8.5)			
-Hypertension, *n* (%)	4 (6.5)			
-Diabetes mellitus, *n* (%)	2 (3)			
-No underlying comorbidity, *n* (%)	29 (48)			
ICU admission diagnoses				
-Acute poisoning, *n* (%)	43 (72)	33 (92)	10 (42)	˂10^−3^
-Acute respiratory failure, *n* (%)	12 (20)	2 (5)	10 (42)	0.004
-Endocrine disorders, *n* (%)	1	0	1	0.4
-Neurologic disease, *n* (%)	4 (7)	2 (5)	2 (8)	1
Gravity scores				
-SAPS II, median [IQR]	40.5 [32–44]	36.5 [32–44]	41 [39–47]	0.049
-APACHE II, median [IQR]	19 [15–22]	17 [15–22]	21 [19–23]	0.002
-SOFA, median [IQR]	6 [5–10]	5.5 [4–10]	8 [6–10]	0.037
-Prior antibiotic use, *n* (%)	41(68)	52	92	0.002
ICU stay				
Duration of ICU stay days, median [IQR]	17 [12–31]			
Duration mechanical ventilation, days, median [IQR]	11 [8–24]			
Sepsis, *n* (%)	7 (12)			
ARDS, *n* (%)	9 (15)			
Mortality, *n* (%)	32 (53)			
Mortality at D14		7 (19)	3 (12)	0.37
Mortality at D28		12 (33)	4 (17)	0.061

SD = standard deviation; COPD = chronic obstructive pulmonary disease, SAPS II = Simplified Acute Physiology Score II, APACHE II = Acute Physiology and Chronic Health Evaluation II; IQR = interquartile range; ICU = intensive care unit; SOFA = Sequential Organ Failure Assessment, MV = mechanical ventilation, ARDS = acute respiratory distress syndrome.

**Table 2:** Etiological agents.

Pathogens	*n* (%)
*A. baumannii*	21 (35)
*P. aeruginosa*	12 (20)
*Enterobacterales*	10 (17)
*S. aureus*	3 (5)
*A. baumannii + P. aeruginosa*	7 (12)
*A. baumannii +* Enterobacterales	4 (6)
*P. aeruginosa +* Enterobacterales	3 (5)

**Table 3:** Organisms isolated from early- and late-onset VAP cases.

Pathogen	Total Isolates *n* (%)	Early-Onset VAP	Late-Onset VAP	*p*
*A. baumannii*	32 (53)	17 (47)	15 (62)	0.3
*P. aeruginosa*	22 (37)	13 (36)	9 (37)	1
*Enterobacterales*	17 (28)	11 (30)	6 (25)	0.77
*S. aureus*	3 (5)	3 (5)	_	0.27
-MSSA	2	2	_	
-MRSA	1	1	_	
Potentially MDR; *n* (%)	58 (97)			

MRSA = methicillin-resistant *S. aureus*, MSSA = methicillin-sensitive S. aureus, MDR: multidrug resistant.

**Table 4:** Etiological agents of VAP and antibacterial resistance pattern.

Pathogens	Antibiotic Resistance Pattern (% of Resistance)														
	AMX	AMC	TIC	TCC	PIP	TZP	CAZ	IMP	GEN	AM	NET	CST	CIP	FOS	RIF
*A. baumannii (n = 32)*	-	-	100	100	100	100	100	100	100	85	26	0	100	85	60
*P. aeruginosa (n = 22)*	-	-	77	55	44	0	67	44	77	11	0	0	48	11	-
Enterobacterales (*n* = 17)	100	71	100	71	100	71	71	0	28	14	28	-	14	-	-

AMX: amoxicillin; AMC: amoxicillin–clavulanic acid, TIC: ticarcillin, TCC: ticarcillin–clavulanic acid, PIP: piperacillin, TZP: pipercillin–tazobactam, CAZ: ceftazidime, IMP: imipenem, GEN: gentamicin, AM: amikacin, NET: netilmycin, CST: colistin, CIP: ciprofloxacin, FOS: fosfomycin, RIF: rifampin; -: not tested.

**Table 5:** Antibacterial resistance pattern of *A. baumannii*, *P. aeruginosa*, Enterobacterales.

Antibiotic	*A. baumannii* (%)		*p*	*P. aeruginosa* (%)		*p*	Enterobacterales (%)		*p*
	Early-Onset VAP (*n* = 17)	Late-Onset VAP (*n* = 15)		Early-Onset VAP (*n* = 13)	Late-Onset VAP (*n* = 9)		Early-Onset VAP (*n* = 11)	Late-Onset VAP (*n* = 6)	
TIC	100	100	-	62	66	1	100	100	-
TCC	100	100	-	48	66	0.4	30	30	
PIP	100	100	-	38	66	0.4	100	100	-
TZP	100	100	-	31	25	1	30	30	
CAZ	100	100	-	54	66	0.7	82	83	
IMP	100	100	-	54	56	1	0	0	-
GEN	88	93	1	75	62	0.6	40	50	0.3
AM	71	82	0.2	23	25	0.6	34	50	0.3
NET	12	33	0.6	11	8	1	10	0	1
CST	0	0	-	100	100	-	0	0	-
CIP	88	86	1	54	67	0.7	40	50	0.3
FOF	0.76	100	0.1	25	28	0.6	40	50	0.3
RIF	0.71	0.47	0.3	85	89	1	75	66	1

TIC: ticarcillin; TCC: ticarcillin–clavulanic acid; PIP: piperacillin; TZP: pipercillin–tazobactam; CAZ: ceftazidime; IMP: imipenem; GEN: gentamicin; AM: amikacin; NET: netilmycin; CST: colistin; CIP: ciprofloxacin; FOS: fosfomycin; RIF: rifampin; -: not tested.

## Data Availability

Data are available upon request.
